# PowerPlay: Training an Increasingly General Problem Solver by Continually Searching for the Simplest Still Unsolvable Problem

**DOI:** 10.3389/fpsyg.2013.00313

**Published:** 2013-06-07

**Authors:** Jürgen Schmidhuber

**Affiliations:** ^1^The Swiss AI Lab IDSIA, University of Lugano, SUPSI, Lugano, Switzerland

**Keywords:** problem discovery, task invention, skill learning, general problem solver, intrinsic motivation, curiosity, creativity

## Abstract

Most of computer science focuses on automatically solving given computational problems. I focus on automatically *inventing* or *discovering* problems in a way inspired by the playful behavior of animals and humans, to train a more and more general problem solver from scratch in an unsupervised fashion. Consider the infinite set of all computable descriptions of tasks with possibly computable solutions. Given a general problem-solving architecture, at any given time, the novel algorithmic framework PowerPlay (Schmidhuber, 2011) searches the space of possible *pairs* of new tasks and modifications of the current problem solver, until it finds a more powerful problem solver that provably solves all previously learned tasks plus the new one, while the unmodified predecessor does not. Newly invented tasks may require to achieve a *wow-effect* by making previously learned skills more efficient such that they require less time and space. New skills may (partially) re-use previously learned skills. The greedy search of typical PowerPlay variants uses time-optimal program search to order candidate pairs of tasks and solver modifications by their conditional computational (time and space) complexity, given the stored experience so far. The new task and its corresponding task-solving skill are those first found and validated. This biases the search toward pairs that can be described compactly and validated quickly. The computational costs of validating new tasks need not grow with task repertoire size. Standard problem solver architectures of personal computers or neural networks tend to generalize by solving numerous tasks outside the self-invented training set; PowerPlay’s ongoing search for novelty keeps breaking the generalization abilities of its present solver. This is related to Gödel’s sequence of increasingly powerful formal theories based on adding formerly unprovable statements to the axioms without affecting previously provable theorems. The continually increasing repertoire of problem-solving procedures can be exploited by a parallel search for solutions to additional externally posed tasks. PowerPlay may be viewed as a greedy but practical implementation of basic principles of creativity (Schmidhuber, 2006a, 2010). A first experimental analysis can be found in separate papers (Srivastava et al., 2012a,b, 2013).

## 1 Introduction

Given a realistic piece of computational hardware with specific resource limitations, how can one devise software for it that will solve all, or at least many, of the *a priori* unknown tasks that are in principle easily solvable on this architecture? In other words, how to build a *practical* general problem solver, given the computational restrictions? It does not need to be *universal* and *asymptotically* optimal (Levin, 1973; Hutter, 2002; Schmidhuber, 2004b, 2009) like the recent (not necessarily practically feasible) general problem solvers discussed in Section 7.2; instead it should take into account all constant architecture-specific slowdowns ignored in the asymptotic optimality notation of theoretical computer science, and be generally useful for real-world applications.

Let us draw inspiration from biology. How do initially helpless human babies become rather general problem solvers over time? Apparently by playing. For example, even in the absence of external reward or hunger they are curious about what happens if they move their eyes or fingers in particular ways, creating little experiments which lead to initially novel and surprising but eventually predictable sensory inputs, while also learning motor skills to reproduce these outcomes. (See Schmidhuber, 1991a,b, 1999, 2006a, 2010; Yi et al., 2011 and Section 7.4 for previous artificial systems of this type.) Infants continually seem to invent new tasks that become boring as soon as their solutions become known. Easy-to-learn new tasks are preferred over unsolvable or hard-to-learn tasks. Eventually the numerous skills acquired in this creative, self-supervised way may get re-used to facilitate the search for solutions to external problems, such as finding food when hungry. While kids keep inventing new problems for themselves, they move through remarkable developmental stages (Harlow et al., 1950; Berlyne, 1954; Piaget, 1955).

Here I introduce a novel unsupervised algorithmic framework for training a computational problem solver from scratch, continually searching for the simplest (fastest to find) combination of task and corresponding task-solving skill to add to its growing repertoire, without forgetting any previous skills (Section 2), or at least without decreasing average performance on previously solved tasks (Section 6.1). New skills may (partially) re-use previously learned skills. Every new task added to the repertoire is essentially defined by the time required to invent it, to solve it, and to demonstrate that no previously learned skills got lost. The search takes into account that typical problem solvers may learn to solve tasks outside the growing self-made training set due to generalization properties of their architectures. The framework is called PowerPlay because it continually (Ring, 1994) aims at boosting computational ability and problem-solving capacity, reminiscent of humans or human societies trying to boost their general power/capabilities/knowledge/skills in playful ways, even in the absence of externally defined goals, although the skills learned by this type of pure curiosity may later help to solve externally posed tasks.

Unlike our first implementations of curious/creative/playful agents from the 1990s (Schmidhuber, 1991a, 1999; Storck et al., 1995) (Section 7.4; compare (Barto, 2013; Dayan, 2013; Nehmzow et al., 2013; Oudeyer et al., 2013)), PowerPlay provably (by design) does not have any problems with online learning – it cannot forget previously learned skills, automatically segmenting its life into a sequence of clearly identified tasks with explicitly recorded solutions. Unlike the task search of theoretically optimal creative agents (Schmidhuber, 2006a, 2010) (Section 7.4), PowerPlay’s task search is greedy, but at least practically feasible.

Some claim that scientists often invent appropriate problems for their methods, rather than inventing methods to solve given problems. The present paper formalizes this in a way that may be more convenient to implement than those of our previous work (Schmidhuber, 1991a, 1999, 2006a, 2010), and describes a simple practical framework for building creative artificial scientists or explorers that by design continually come up with the fastest to find, initially novel, but eventually solvable problems.

### 1.1 Basic ideas

In traditional computer science, given some formally defined task, a search algorithm is used to search a space of solution candidates until a solution to the task is found and verified. If the task is hard the search may take long.

To automatically construct an increasingly general problem solver, let us expand the traditional search space in an unusual way, such that it includes all possible *pairs* of computable tasks with possibly computable solutions, and problem solvers. Given an old problem solver that can already solve a finite known set of previously learned tasks, a search algorithm is used to find a new pair that provably has the following properties: (1) the new task cannot be solved by the old problem solver. (2) The new task can be solved by the new problem solver (some modification of the old one). (3) The new solver can still solve the known set of previously learned tasks.

Once such a pair is found, the cycle repeats itself. This will result in a continually growing set of known tasks solvable by an increasingly more powerful problem solver. Solutions to new tasks may (partially) re-use solutions to previously learned tasks.

Smart search (e.g., Section 4.1 and Algorithm 4.1) orders candidate pairs of the type (*task, solver*) by computational complexity, using concepts of optimal universal search (Levin, 1973; Schmidhuber, 2004b), with a bias toward pairs that can be described by few additional bits of information (given the experience so far) and that can be validated quickly.

At first glance it might seem harder to search for pairs of tasks and solvers instead of solvers only, due to the apparently larger search space. However, the additional freedom of *inventing* the tasks to be solved may actually greatly reduce the time intervals between problem solver advances, because the system may often have the option of inventing a rather simple task with an easy-to-find solution.

A new task may be about simplifying the old solver such that it can still solve all tasks learned so far, but with less computational resources such as time and storage space (e.g., Section 3.1 and Algorithm 6.1).

Since the new pair (*task, solver*) is the first one found and validated, the search automatically trades off the time-varying efforts required to either invent completely new, previously unsolvable problems, or compressing/speeding up previous solutions. Sometimes it is easier to refine or simplify known skills, sometimes to invent new skills.

On typical problem solver architectures of personal computers (PCs) or neural networks (NNs), while a limited known number of previously learned tasks has become solvable, so too has a large number of unknown, never-tested tasks (in the field of Machine Learning, this is known as *generalization*). PowerPlay’s ongoing search is continually testing (and always trying to go beyond) the generalization abilities of the most recent solver instance; some of its search time has to be spent on demonstrating that self-invented new tasks are not already solvable.

Often, however, much more time will have to be spent on making sure that a newly modified solver did not forget any of the possibly many previously learned skills. Problem solver modularization (Section 3.3, especially 3.3.2) may greatly reduce this time though, making PowerPlay prefer pairs whose validation does not require the re-testing of too many previously learned skills, thus decomposing at least part of the search space into somewhat independent regions, realizing *divide and conquer* strategies as by-products of its built-in drive to invent and validate novel tasks/skills as quickly as possible.

A biologically inspired hope is that as the problem solver is becoming more and more general, it will find it easier and easier to solve externally posed tasks (Section 5), just like growing infants often seem to re-use their playfully acquired skills to solve teacher-given problems.

### 1.2 Outline of remainder

Section 2 will introduce basic notation and Variant 1 of the algorithmic framework PowerPlay, which invokes the essential procedures Task Invention, Solver Modification, and Correctness Demonstration. Section 3 will discuss details of these procedures.

More detailed instantiations of PowerPlay will be described in Section 4.3 (an evolutionary method, Algorithm 4.3) and Section 4.1 (an asymptotically optimal program search method, Algorithm 4.1).

As mentioned above, the skills acquired to solve self-generated tasks may later greatly facilitate solutions to externally posed tasks, just like the numerous motor skills learned by babies during curious exploration of its world often can be re-used later to maximize external reward. Sections 5 and 6.1 will discuss variants of the framework (e.g., Algorithm 6.1) in which some of the tasks can be defined externally.

Section 6.1 will also describe a natural variant of the framework that explicitly penalizes solution costs (including time and space complexity), and allows for forgetting aspects of previous solutions, provided the average performance on previously solved tasks does not decrease.

Section 7 will point to illustrative experiments (Section 7.8) described in separate papers (Srivastava et al., 2012b, 2013), and discuss relations to previous work.

## 2 Notation and Algorithmic Framework PowerPlay (Variant I)

B* denotes the set of finite sequences or bitstrings over the binary alphabet *B*  = {0, 1}, λ the empty string, *x*, *y*, *z*, *p*, *q*, *r*, *u* strings in B*, 𝒩 the natural numbers, ℝ the real numbers, ∊ ∈ ℝ a positive constant, *m*, *n*, *n*_0_, *k*, *i*, *j*, *k*, *l* non-negative integers, *L*(*x*) the number of bits in *x* (where *L*(λ) = 0), *f*, *g* functions mapping integers to integers. We write *f*  (*n*) = *O*(*g*(*n*)) if there exist positive *c*, *n*_0_ such that *f* (*n*) ≤ *cg*(*n*) for all *n*  > *n*_0_.

The computational architecture of the problem solver may be a deterministic universal computer, or a more limited device such as a finite state automaton or a feedforward neural network (NN) (Bishop, 2006). All such problem solvers can be uniquely encoded (Gödel, 1931) or implemented on universal computers (Church, 1936; Post, 1936; Turing, 1936) such as universal Turing Machines (TM). Therefore, without loss of generality, the remainder of this paper assumes a fixed universal reference computer whose input programs and outputs are elements of B*. A user-defined subset 𝒮 ⊂ B* defines the set of possible problem solvers. For example, if the problem solver’s architecture is itself a binary universal TM or a standard computer, then 𝒮represents its set of possible programs, or a limited subset thereof – compare Section 3.2 and 4.1. If it is a feedforward NN, then 𝒮 could be a highly restricted subset of programs encoding the NN’s possible topologies and weights (floating point numbers) – compare Section 7.8 and the original SLIM NN paper (Schmidhuber, 2012).

In what follows, for convenience I will often identify bitstrings in B* with things they encode, such as integers, real-valued vectors, weight matrices, or programs – the context will always make clear what is meant.

The problem solver’s initial program is called *s*_0_. There is a set of possible task descriptions 𝒯 ⊂ B*. 𝒯may be the infinite set of *all* possible computable descriptions of tasks with possibly computable solutions, or just a small subset thereof. For example, a simple task may require the solver to answer a particular input pattern with a particular output pattern (more formal details on pattern recognition tasks are given in Section 3.1.1). Or it may require the solver to steer a robot toward a goal through a sequence of actions (more formal details on sequential decision-making tasks in unknown environments are given in Section 3.1.2). There is a particular sequence of task descriptions *T*_1_, *T*_2_, …, where each unique *T_i_* ∈ 𝒯 (*i* = 1, 2, …) is chosen or “invented” by a search method described below such that the solutions of *T*_1_, *T*_2_, …, *T_i_* can be computed by *s_i_*, the *i*-th instance of the program, but not by *s*_*i*−1_ (*i*  = 1, 2, …). Each *T_i_* consists of a unique problem identifier that can be read by *s_i_* through some built-in input processing mechanism (e.g., input neurons of an NN (Schmidhuber, 2012)), and a unique description of a deterministic procedure for determining whether the problem has been solved. Denote *T*_≤*i*_ = {*T*_1_, …, *T_i_*}; *T*_<*i*_ = {*T*_1_, …, *T_i_−1*}.

A valid task *T_i_*(*i*  > 1) may require solving at least one previously solved task *T_k_*(*k*  < *i*) more efficiently, by using less resources such as storage space, computation time, energy, etc., thus achieving a *wow-effect*. See Section 3.1.

Tasks and problem solver modifications are computed and validated by elements of another appropriate set of programs 𝒫 ⊂ B*. Programs *p* ∈ 𝒫 may contain instructions for reading and executing (parts of) the code of the present problem solver and reading (parts of) a recorded history *Trace* ∈ B* of previous events that led to the present solver. The algorithmic framework (Algorithm 2) incrementally trains the problem solver by finding *p* ∈ 𝒫 that increase the set of solvable tasks.

## 3 Task Invention, Solver Modification, Correctness Demo

A program tested by Algorithm 2 has to allocate its runtime to solve three main jobs, namely, Task Invention, Solver Modification, Correctness Demonstration. Now examples of each will be listed.

### 3.1 Implementing Task Invention

Part of the job of *p_i_* ∈ 𝒫 is to compute *T_i_* ∈ 𝒯. This will consume some of the total computation time allocated to *p_i_*. Two examples will be given: pattern recognition tasks are treated in Section 3.1.1; sequential decision-making tasks in Section 3.1.2.

**Algorithm 2: Algorithmic Framework** PowerPlay **(Variant I)**



Initialize *s*_0_ in some way.
**for** *i*: = 1, 2, …**do**
 **repeat**
  Let a search algorithm (examples in Section 4) create a new candidate program *p* ∈ 𝒫.
  Give *p* limited time to do (not necessarily in this order):
  * Task Invention: Let *p* compute a task *T* ∈ 𝒯. See Section 3.1.
  * Solver Modification: Let *p* compute a value of the variable *q* ∈ 𝒮 ⊂ B* (a candidate for *s_i_*)
  by computing a modification of *s*_*i*−1_. See Section 3.2.
  * Correctness Demonstration: Let *p* try to show that *T* cannot be solved by *s*_*i*−1_, but that
  *T* and all *T_k_*(*k < i*) can be solved by *q*. See Section 3.3.
 **until** Correctness Demonstration was successful
 Set *p_i_*: = *p*;*T_i_*: = *T*;*s_i_*: = *q*; update *Trace*.
**end for**



#### 3.1.1 Example: pattern recognition tasks

In the context of learning to recognize or analyze patterns, *T_i_* could be a 4-tuple (*I_i_*, *O_i_*, *t_i_*, *n_i_*) ∈ ℐ × 𝒪 × ℕ × ℕ, where ℐ, 𝒪 ⊂ ℬ*, and *T _i_* is solved if *s_i_* satisfies *L*(*s_i_*) < *n_i_* and needs at most *t_i_* discrete time steps to read *I_i_* and compute *O_i_* and halt. Here *I_i_* itself may be a pair $\left(I_{i}^{1},I_{i}^{2}\right)\in B^{*}\times B^{*}$, where ${\text{I}}_{i}^{1}$ is constrained to be the address of an image in a given database of patterns, and ${\text{I}}_{i}^{2}$ is a *p_i_*-generated “*query*” that uniquely specifies *how* the image should be classified through target pattern *O_i_*, such that the same image can be analyzed in different ways during different tasks. For example, depending on the nature of the invented task sequence, the problem solver could eventually learn that *O*  = 1 if *I*^2^ = 1001 (suppressing task indices) and the image addressed by *I*^1^ contains at least one black pixel, or if *I*^2^ = 0111 and the image shows a cow.

Since the definition of task *T_i_* includes bounds *n_i_*, *t_i_* on computational resources, *T_i_* may be about solving at least one *T_k_*(*k*  < *i*) more efficiently, corresponding to a *wow-effect*. This in turn may also yield more efficient solutions to other tasks *T_l_*(*l*  < *i*, *l* ≠ *k*). In practical applications one may insist that such efficiency gains must exceed a certain threshold ε  > 0, to avoid task series causing sequences of very minor improvements.

Note that *n_i_* and *t_i_* may be unnecessary in special cases such as the problem solver being a fixed topology feedforward NN (Bishop, 2006) whose input and target patterns have constant size and whose computational efforts per pattern need constant time and space resources.

Assuming sufficiently powerful 𝒮, 𝒫, in the beginning, trivial tasks such as simply copying $I_{i}^{2}$ onto *O_i_* may be interesting in the sense that PowerPlay can still validate and accept them, but they will become boring (inadmissible) as soon as they are solvable by solutions to previous tasks that generalize to new tasks.

#### 3.1.2 Example: general decision-making tasks in dynamic environments

In the more general context of general problem solving/sequential decision making/reinforcement learning/reward optimization (Newell and Simon, 1963; Kaelbling et al., 1996; Sutton and Barto, 1998) in unknown environments, there may be a set ℐ ⊂ B* of possible task identification patterns and a set 𝒥 ⊂ B*of programs that test properties of bitstrings. *T _i_* could then encode a 4-tuple (*I_i_*, *J_i_*, *t_i_*, *n_i_*) ∈ ℐ × 𝒥 × ℕ × ℕ of finite bitstrings with the following interpretation: *s_i_* must satisfy *L*(*s_i_*) < *n_i_* and may spend at most *t_i_* discrete time steps on first reading *I_i_* and then interacting with an environment through a sequence of perceptions and actions, to achieve some computable goal defined by *J_i_*.

More precisely, while *T_i_* is being solved within *t_i_* time steps, at any given time 1 ≤ *t*  ≤ *t_i_*, the internal state of the problem solver at time *t* is denoted *u_i_*(*t*) ∈ B*; its initial default value is *u_i_*(0). For example, *u_i_*(*t*) may encode the current contents of the internal tape of a TM, or of certain addresses in the dynamic storage area of a PC, or the present activations of an LSTM recurrent NN (Hochreiter and Schmidhuber, 1997). At time *t*, *s_i_* can spend a constant number of elementary computational instructions to copy the task description *T_i_* or the present environmental input *x_i_*(*t*) ∈ B* and a reward signal *r_i_*(*t*) ∈ B* (interpreted as a real number) into parts of *u_i_*(*t*), then update other parts of *u_i_*(*t*) (a function of *u_i_*(*t* − 1)) and compute action *y_i_*(*t*) ∈ B* encoded as a part of *u_i_*(*t*). *y_i_*(*t*) may affect the environment, and thus future inputs.

If 𝒫 allows for programs that can *dynamically acquire additional physical computational resources* such as additional CPUs and storage, then the above constant number of elementary computational instructions should be replaced by a constant amount of real time, to be measured by a reliable physical clock.

The sequence of 4-tuples (*x_i_*(*t*), *r_i_*(*t*), *u_i_*(*t*), *y_i_*(*t*)) (*t*  = 1, …, *t_i_*) gets recorded by the so-called trace *Trace_i_* ∈ B*. If at the end of the interaction a desirable computable property *J_i_*(*Trace_i_*) (computed by applying program *J_i_* to *Trace_i_*) is satisfied, then by definition the task is solved. The set 𝒥 of possible *J_i_* may represent an infinite set of all computable tasks with solutions computable by the given hardware. For practical reasons, however, the set 𝒥 of possible *J_i_* may also be restricted to bit sequences encoding just a few possible goals. For example, *J_i_* may only encode goals of the form: a robot arm steered by program or “*policy*” *s_i_* has reached a certain target (a desired final observation *x_i_*(*t_i_*) recorded in *Trace_i_*) without measurably bumping into an obstacle along the way, that is, there were no negative rewards, that is, *r_i_*(τ) ≥ 0 for τ  = 1 … *t_i_*.

If the environment is deterministic, e.g., a digital physics simulation of a robot, then its current state can be encoded as part of *u*(*t*), and it is straight-forward for Correctness Demonstration to test whether some *s_i_* still can solve a previously solved task *T_j_*(*j*  < *i*). *However, what if the environment is only partially observable, like the real world, and non-stationary, changing in unknown ways?* Then Correctness Demonstration must check whether *s_i_* still produces the same action sequence in response to the input sequence recorded in *Trace_j_* (often this replay-based test will actually be computationally cheaper than a test involving the environment). *Achieving the same goal in a changed environment must be considered a different task, even if the changes are just due to noise on the environmental inputs*. (Sure, in the real world *s_j_*(*j*  > *i*) might actually achieve *J_i_* faster than *s_i_*, given the description of *T_i_*, but Correctness Demonstration in general cannot know whether this acceleration was due to plain luck – it must stick to reproducing *Trace_j_* to make sure it did not forget anything.)

See Section 6.2, however, for a less strict PowerPlay variant whose Correctness Demonstration directly interacts with the real world to collect sufficient problem-solving statistics through repeated trials, making certain assumptions about the probabilistic nature of the environment, and the repeatability of experiments.

### 3.2 Implementing Solver Modification

Part of the job of *p_i_* ∈ 𝒫 is also to compute *s_i_*, possibly profiting from having access to *s_i_*_−1_, because only few changes of *s_i_*_−1_ may be necessary to come up with an *s_i_* that goes beyond *s_i_*_−1_. For example, if the problem solver is a standard PC, then just a few bits of the previous software *s_i_*_−1_ may need to be changed.

For practical reasons, the set 𝒮 of possible *s_i_* may be greatly restricted to bit sequences encoding programs that obey the syntax of a standard programing language such as LISP or Java. In turn, the programing language describing 𝒫 should be greatly restricted such that any *p_i_* ∈ 𝒫 can only produce syntactically correct *s_i_*.

If the problem solver is a feedforward NN with pre-wired, unmodifiable topology, then 𝒮 will be restricted to those bit sequences encoding valid weight matrices, *s_i_* will encode its *i*-th weight matrix, and 𝒫 will be restricted to those *p* ∈ 𝒫 that can produce some *s_i_* ∈ 𝒮. Depending on the user-defined programing language, *p_i_* may invoke complex pre-wired subprograms (e.g., well-known learning algorithms) as primitive instructions – compare separate experimental analysis (Srivastava et al., 2012b, 2013).

In general, *p* itself determines how much time to spend on Solver Modification – enough time must be left for Task Invention and Correctness Demonstration.

### 3.3 Implementing Correctness Demonstration

Correctness demonstration may be the most time-consuming obligation of *p_i_*. At first glance it may seem that as the sequence *T*_1_, *T*_2_, … is growing, more and more time will be needed to show that *s_i_* but not *s*_*i*−1_ can solve *T*_1_, *T*_2_, …, *T_i_*, because one naive way of ensuring correctness is to re-test *s_i_* on all previously solved tasks. Theoretically more efficient ways are considered next.

#### 3.3.1 Most general: proof search

The most general way of demonstrating correctness is to encode (in read-only storage) an axiomatic system 𝒜 that formally describes computational properties of the problem solver and possible *s_i_*, and to allow *p_i_* to search the space of possible proofs derivable from 𝒜, using a proof searcher subroutine that systematically generates proofs until it finds a theorem stating that *s_i_* but not *s*_*i*−1_ solves *T*_1_, *T*_2_, …, *T_i_* (proof search may achieve this efficiently without explicitly re-testing *s_i_* on *T*_1_, *T*_2_, …, *T_i_*). This could be done like in the Gödel Machine (Schmidhuber, 2009) (Section 7.2), which uses an online extension of *Universal Search* (Levin, 1973) to systematically test *proof techniques*: proof-generating programs that may invoke special instructions for generating axioms and applying inference rules to prolong an initially empty *proof*  ∈ B* by theorems, which are either axioms or inferred from previous theorems through rules such as *modus ponens* combined with *unification*, e.g., (Fitting, 1996). 𝒫 can be easily limited to programs generating only syntactically correct proofs (Schmidhuber, 2009). 𝒜 has to subsume axioms describing how any instruction invoked by some *s* ∈ 𝒮 will change the state *u* of the problem solver from one step to the next (such that proof techniques can reason about the effects of any *s_i_*). Other axioms encode knowledge about arithmetics etc (such that proof techniques can reason about spatial and temporal resources consumed by *s_i_*).

In what follows, Correctness Demonstrations will be discussed that are less general but sometimes more convenient to implement.

#### 3.3.2 Keeping track which components of the solver affect which tasks

Often it is possible to partition *s* ∈ 𝒮 into components, such as individual bits of the software of a PC, or weights of a NN. Here the *k*-th component of *s* is denoted *s^k^*. For each *k* (*k*  = 1, 2, …) a variable list $L^{k}=\left(T_{1}^{k},T_{2}^{k},\ldots \right)$ is introduced. Its initial value before the start of PowerPlay is $L_{0}^{k}$, an empty list. Whenever *p_i_* found *s_i_* and *T_i_* at the end of Correctness Demonstration, each *L^k^* is updated as follows: its new value $L_{i}^{k}$ is obtained by appending to $L_{i-1}^{k}$ those $T_{j}\notin L_{i-1}^{k}\left(j=1,\ldots ,i\right)$ whose current (possibly revised) solutions now need *s^k^* at least once during the solution-computing process, and deleting those *T_j_* whose current solutions do not use *s^k^* any more.

PowerPlay’s Correctness Demonstration thus has to test only tasks in the union of all $L_{i}^{k}$. That is, if the most recent task does not require changes of many components of *s*, and if the changed bits do not affect many previous tasks, then Correctness Demonstration may be very efficient.

Since every new task added to the repertoire is essentially defined by the time required to invent it, to solve it, and to show that no previous tasks became unsolvable in the process, PowerPlay is generally “motivated” to invent tasks whose validity check does not require too much computational effort. That is, PowerPlay will often find *p_i_* that generate *s_i_*_−1_-modifications that don’t affect too many previous tasks, thus decomposing at least part of the spaces of tasks and their solutions into more or less independent regions, realizing *divide and conquer* strategies as by-products. Compare a recent experimental analysis of this effect (Srivastava et al., 2012b, 2013).

#### 3.3.3 Advantages of prefix code-based problem solvers

Let us restrict 𝒫 such that tested *p* ∈ 𝒫 cannot change any components of *s_i_*_−1_ during Solver Modification, but can create a new *s_i_* only by adding new components to *s_i_*_−1_. (This means freezing all used components of any *s_k_* once *T_k_* is found.) By restricting *S* to self-delimiting prefix codes like those generated by the Optimal Ordered Problem Solver (OOPS) (Schmidhuber, 2004b), one can now profit from a sometimes particularly efficient type of Correctness Demonstration, ensuring that differences between *s_i_* and *s_i_*_−1_ cannot affect solutions to *T*_<*i*_ under certain conditions. More precisely, to obtain *s_i_*, half the search time is spent on trying to process *T_i_* first by *s_i_*_−1_, extending or prolonging *s_i_*_−1_ only when the ongoing computation requests to add new components through special instructions (Schmidhuber, 2004b) – then Correctness Demonstration has less to do as the set *T*_<*i*_ is guaranteed to remain solvable, by induction. The other half of the time is spent on processing *T_i_* by a new sub-program with new components $s\prime_{i}$, a part of *s_i_* but *not* of *s_i_*_−1_, where $s\prime_{i}$ may read *s*_*i*−1_ or invoke parts of *s_i_*_−1_ as sub-programs to solve *T*_≤*i*_ – only then Correctness Demonstration has to test *s_i_* not only on *T_i_* but also on *T*_<*i*_ (see (Schmidhuber, 2004b) for details).

A simple but not very general way of doing something similar is to interleave Task Invention, Solver Modification, Correctness Demonstration as follows: restrict all *p* ∈ 𝒫 such that they must define *I_i_*: = *i* as the unique task identifier *I_i_* for *T_i_* (see Section 3.1.2); restrict all *s* ∈ 𝒮 such that the input of *I_i_* = *i* automatically invokes sub-program $s\prime_{i}$, a part of *s_i_* but *not* of *s_i_*_−1_ (although $s\prime_{i}$ may read *s_i_*_−1_ or invoke parts of *s_i_*_−1_ as sub-programs to solve *T_i_*). Restrict *J_i_* to a subset of acceptable computational outcomes (Section 3.1.2). Run *s_i_* until it halts and has computed a *novel* output acceptable by *J_i_* that is different from all outputs computed by the (halting) solutions to *T*_<*i*_; this novel output becomes *T_i_* ’s goal. By induction over *i*, since all previously used components of *s_i_*_−1_ remain unmodified, the set *T*_<*i*_ is guaranteed to remain solvable, no matter $s\prime_{i}$. That is, Correctness Demonstration on previous tasks becomes trivial. However, in this simple setup there is no immediate generalization across tasks like in OOPS (Schmidhuber, 2004b) and the previous paragraph: the trivial task identifier *i* will always first invoke some $s\prime_{i}$ different from all $s\prime_{k}\left(k\neq i\right)$, instead of allowing for solving a new task solely by previously found code.

## 4 Implementations of PowerPlay

PowerPlay is a general framework that allows for plugging in many differents search and learning algorithms. The present section will discuss some of them.

### 4.1 Implementation based on optimal ordered problem solver OOPS

The *i*-th problem is to find a program *p_i_* ∈ 𝒫 that creates *s_i_* and *T_i_* and demonstrates that *s_i_* but not *s_i_*_−1_ can solve *T*_1_, *T*_2_, …, *T_i_*. This yields a perfectly ordered problem sequence for a variant of the *Optimal Ordered Problem Solver* OOPS (Schmidhuber, 2004b) (Algorithm 4.1).

**Algorithm 4.1: Implementing** PowerPlay **with Procedure OOPS** (Schmidhuber, 2004b)



(see text for details) - initialize *s*_0_ and *u* (internal dynamic storage for *s*  ∈ 𝒮) and *U* (internal dynamic storage for *p* ∈ 𝒫),
where each possible *p* is a sequence of subprograms *p*’, *p*”, *p*”’.
**for** *i*:= 1, 2, …**do**
 set variable time limit *t_lim_*:= 1;
 let the variable set *H* be empty;
 set Boolean variable DONE: = FALSE
 **repeat**
  **if** *H* is empty **then**
   set *t_lim_* : = 2*t_lim_*; *H* : = {*p* ∈ 𝒫: *P*(*p*)*t_lim_* ≥ 1}
  **else**
   choose and remove some *p* from *H*
   **while** not DONE and less than *P*(*p*)*t_lim_* time was spent on *p* **do**
    execute the next time step of the following computation:
    1. Let *p*’ compute some task *T*  ∈ 𝒯 and halt.
    2. Let *p*” compute *q*  ∈ 𝒮 by modifying a copy of *s*_*i*−1_, and halt.
    3. Let *p*”’ try to show that *q* but not *s_i_*_-1_ can solve *T*_1_, *T*_2_, …, *T_i_*_-1_, *T*.
     If *p*”’ was successful set DONE:=TRUE.
   **end** **while**
   Undo all modifications of *u* and *U* due to *p*. This does not cost more time than executing *p* in the
   while loop above (Schmidhuber, 2004b).
  **end if**
 **until** DONE
 set *p_i_*:=*p*; *T_i_*:=*T*; *s_i_*:=*q*;
 add a unique encoding of the 5-tuple (*i*, *p_i_*, *s_i_*, *T_i_*, *Trace_i_*) to read-only storage
 readable by programs to be tested in the future.
 **end for**



While a candidate program *p* ∈ 𝒫 is executed, at any given discrete time step *t*  = 1, 2, …, its internal state or dynamical storage *U* at time *t* is denoted *U*(*t*) ∈ B* (not to be confused with the solver’s internal state *u*(*t*) of Section 3.1.2). Its initial default value is *U*(0). E.g., *U*(*t*) could encode the current contents of the internal tape of a TM (to be modified by *p*), or of certain cells in the dynamic storage area of a PC.

Once *p_i_* is found, *p_i_*, *s_i_*, *T_i_*, *Trace_i_* (if applicable; see Section 3.1.2) will be saved in unmodifiable read-only storage, possibly together with other data observed during the search so far. This may greatly facilitate the search for *p_k_*, *k*  > *i*, since *p_k_* may contain instructions for addressing and reading *p_j_*, *s_j_*, *T_j_*, *Trace_j_*(*j*  = 1, …, *k* − 1) and for copying the read code into *modifiable* storage *U*, where *p_k_* may further edit the code, and execute the result, which may be a useful subprogram (Schmidhuber, 2004b).

Define a probability distribution *P*(*p*) on 𝒫 to represent the searcher’s initial bias (more likely programs *p* will be tested earlier (Levin, 1973)). *P* could be based on program length, e.g., *P*(*p*) = 2^−*L*(*p*)^, or on a probabilistic syntax diagram (Schmidhuber, 2004a,b). See Algorithm 4.1.

OOPS keeps doubling the time limit until there is sufficient runtime for a sufficiently likely program to compute a novel, previously unsolvable task, plus its solver, which provably does not forget previous solutions. OOPS allocates time to programs according to an asymptotically optimal universal search method (Levin, 1973) for problems with easily verifiable solutions, that is, solutions whose validity can be quickly tested. Given some problem class, if some unknown optimal program *p* requires *f*  (*k*) steps to solve a problem instance of size *k* and demonstrate the correctness of the result, then this search method will need at most *O*(*f*  (*k*)*/P*(*p*)) = *O*(*f*  (*k*)) steps – the constant factor 1*/P*(*p*) may be large but does not depend on *k*. Since OOPS may re-use previously generated solutions and solution-computing programs, however, it may be possible to greatly reduce the constant factor associated with plain universal search (Schmidhuber, 2004b).

The big difference to previous implementations of OOPS is that PowerPlay has the additional freedom to define its own tasks. As always, every new task added to the repertoire is essentially defined by the time required to invent it, to solve it, and to demonstrate that no previously learned skills got lost.

#### 4.1.1 Building on existing OOPS source code

Existing OOPS source code (Schmidhuber, 2004a) uses a FORTH-like universal programing language to define 𝒫. It already contains a framework for testing new code on previously solved tasks, and for efficiently undoing all *U*-modifications of each tested program. The source code requires few changes to implement the additional task search described above.

#### 4.1.2 Alternative problem solvers based on recurrent neural networks

Recurrent NNs (RNNs, e.g., (Robinson and Fallside, 1987; Werbos, 1988; Schmidhuber, 1992a; Williams and Zipser, 1994; Hochreiter and Schmidhuber, 1997)) are general computers that allow for both sequential and parallel computations, unlike the strictly sequential FORTH-like language of Section 4.1.1. They can compute any function computable by a standard PC (Schmidhuber, 1990). The original PowerPlay report (Schmidhuber, 2011) used a fully connected RNN called RNN1 to define 𝒮, where *w^lk^* is the real-valued *weight* on the directed connection between the *l*-th and *k*-th neuron. To program RNN1 means to set the weight matrix *s*  = ⟨*w^lk^*⟩. Given enough neurons with appropriate activation functions and an appropriate ⟨*w^lk^*⟩, Algorithm 4.1 can be used to train *s*. 𝒫may itself be the set of weight matrices of a separate RNN called RNN2, computing tasks for RNN1, and modifications of RNN1, using techniques for network-modifying networks as described in previous work (Schmidhuber, 1992b, 1993a,b).

In first experiments (Srivastava et al., 2012b, 2013), a particularly suited NN called a self-delimiting NN or SLIM NN (Schmidhuber, 2012) is used. During program execution or activation spreading in the SLIM NN, lists are used to trace only those neurons and connections used at least once. This also allows for efficient resets of large NNs which may use only a small fraction of their weights per task. Unlike standard RNNs, SLIM NNs are easily combined with techniques of asymptotically optimal program search (Levin, 1973; Schmidhuber et al., 1997; Schmidhuber, 2003, 2004b) (Section 4.1). To address overfitting, instead of depending on pre-wired regularizers and hyper-parameters (Bishop, 2006), SLIM NNs can in principle learn to select by themselves their own runtime and their own numbers of free parameters, becoming fast and *slim* when necessary. Efficient SLIM NN learning algorithms (LAs) track which weights are used for which tasks (Section 3.3.2), to greatly speed up performance evaluations in response to limited weight changes. LAs may penalize the task-specific total length of connections used by SLIM NNs implemented on the 3-dimensional brain-like multi-processor hardware to be expected in the future. This encourages SLIM NNs to solve many subtasks by subsets of neurons that are physically close (Schmidhuber, 2012).

### 4.2 Adapting the probability distribution on programs

A straight-forward extension of the above works as follows: whenever a new *p_i_* is found, *P* is updated to make either only *p_i_* or all *p*_1_, *p*_2_, …, *p_i_* more likely. Simple ways of doing this are described in previous work (Schmidhuber et al., 1997). This may be justified to the extent that future successful programs turn out to be similar to previous ones.

### 4.3 Implementation based on stochastic or evolutionary search

A possibly simpler but less general approach is to use an evolutionary algorithm to produce an *s*-modifying and task-generating program *p* as requested by PowerPlay, according to Algorithm 4.3, which refers to the recurrent net problem solver of Section 4.1.2.

**Algorithm 4.3:** PowerPlay **for RNNs Using Stochastic or Evolutionary Search**



Randomly initialize RNN1’s variable weight matrix 〈*w^lk^*〉 and use the result as *s*_0_ (see Section 4.1.2)
**for** *i:=* 1, 2, …**do**
 set Boolean variable DONE = FALSE
 **repeat**
  use a black box optimization algorithm BBOA (many are possible (Rechenberg, 1971; Gomez et al., 2008; Wierstra et al., 2008;   Sehnke et al., 2010)) with adaptive parameter vector  θ  to create some *T*  ∈ 𝒯 (to define the task input to RNN1; see Section 3.1)
  and a modification of *s*_*i*−1_, the current 〈*w^lk^*〉 of RNN1, thus obtaining a new candidate *q* ∈ 𝒮
  **if** *q* but not *s*_*i*−1_ can solve *T* and all *T_k_*(*k ¡ i*) (see Sections 3.3, 3.3.2) **then**
   set DONE = TRUE
  **end if**
 **until** DONE
 set *s_i_*:= *q*; 〈*w^lk^*〉 := *q*; *T_i_*:= *T*; (also store *Trace_i_* if applicable, see Section 3.1.2). Use the information stored so far to adapt the
 parameters  θ of the BBOA, e.g., by gradient-based search (Wierstra et al., 2008; Sehnke et al., 2010), or according to the principles of
 evolutionary computation (Rechenberg, 1971; Gomez et al., 2008; Wierstra et al., 2008).
**end for**



## 5 Adding External Tasks

The growing repertoire of the problem solver may facilitate learning of solutions to externally posed tasks. For example, one may modify PowerPlay such that for certain *i*, *T_i_* is defined externally, instead of being invented by the system itself. In general, the resulting *s_i_* will contain an externally inserted bias in form of code that will make some future self-generated tasks easier to find than others. It should be possible to push the system in a human-understandable or otherwise useful direction by regularly inserting appropriate external goals. See Algorithm 6.1.

Another way of exploiting the growing repertoire is to simply copy *s_i_* for some *I* and use it as a starting point for a search for a solution to an externally posed task *T, without* insisting that the modified *s_i_* also can solve *T*_1_, *T*_2_, …, *T_i_*. This may be much faster than trying to solve *T* from scratch, to the extent the solutions to self-generated tasks reflect general knowledge (code) re-usable for *T*.

In general, however, it will be possible to design external tasks whose solutions do *not* profit from those of self-generated tasks – the latter even may turn out to slow down the search.

On the other hand, in the real world the benefits of curious exploration seem obvious. One should analyze theoretically and experimentally under which conditions the creation of self-generated tasks can accelerate the solution to externally generated tasks – see (Schmidhuber, 1991a, 1999, 2002; Storck et al., 1995; Cuccu et al., 2011; Luciw et al., 2011; Schaul et al., 2011; Yi et al., 2011) for previous simple experimental studies in this vein.

### 5.1 Self-reference through novel task search as an external task

PowerPlay’s *i*-th goal is to find a *p_i_* ∈ 𝒫 that creates *T_i_* and *s_i_* (a modification of *s_i_*_−1_) and shows that *s_i_* but not *s_i_*_−1_ can solve *T*_≤*i*_. As *s* itself is becoming a more and more general problem solver, *s* may help in many ways to achieve such goals in self-referential fashion. For example, the old solver *s_i_*_−1_ may be able to read a unique formal description (provided by *p_i_*) of PowerPlay’s *i*-th goal, viewing it as an external task, and produce an output unambiguously describing a candidate for (*T_i_*, *s_i_*). If *s* has a theorem prover component (Section 3.3.1), *s_i_*_−1_ might even output a full proof of (*T_i_*, *s_i_*)’s validity; alternatively *p_i_* could just use the possibly suboptimal suggestions of *s_i_*_−1_ to narrow down and speed up the search. This is one of the reasons why Section 2 already mentioned that programs *p* ∈ 𝒫 should contain instructions for reading (and running) the code of the present problem solver.

## 6 Softening Task Acceptance Criteria of PowerPlay

The PowerPlay variants above insist that *s* may not solve new tasks at the expense of forgetting to solve any previously solved task within its previously established time and space bounds. For example, let us consider the sequential decision-making tasks from Section 3.1.2. Suppose the problem solver can already solve task *T_k_* = (*I_k_*, *J_k_*, *t_k_*, *n_k_*) ∈ ℐ × 𝒥 × ℕ × ℕ. A very similar but admissible new task *T_i_* = (*I_k_*, *J_k_*, *t_i_*, *n_k_*), (*i*  > *k*), would be to solve *T_k_* substantially faster: *t_i_* < *t_k_* – ε, as long as *T_i_* is not already solvable by *s_i_*_−1_, and no solution to some *T_l_*(*l*  < *i*) is forgotten in the process.

Here I discuss variants of PowerPlay that soften the acceptance criteria for new tasks in various ways, for example, by allowing some of the computations of solutions to previous non-external (Section 5) tasks to slow down by a certain amount of time, provided the *sum* of their runtimes does not increase. This also permits the system to invent new previously unsolved tasks at the expense of slightly increasing time bounds for certain already solved non-external tasks, but without decreasing the average performance on the latter. Of course, PowerPlay has to be modified accordingly, updating average runtime bounds when necessary.

Alternatively, one may allow for trading off space and time constraints in reasonable ways, e.g., in the style of asymptotically optimal *Universal Search* (Levin, 1973), which essentially trades one bit of additional space complexity for a runtime speedup factor of 2.

### 6.1 PowerPlay variant II: Explicitly penalizing time and space complexity

Let us remove time and space bounds from the task definitions of Section 3.1.2, since the modified cost-based PowerPlay framework below (Algorithm 6.1) will handle computational costs (such as time and space complexity of solutions) more directly. In the present section, *T_i_* encodes a tuple (*I_i_*, *J_i_*) ∈ ℐ × 𝒥with interpretation: *s_i_* must first read *I_i_* and then interact with an environment through a sequence of perceptions and actions, to achieve some computable goal defined by *J_i_* within a certain maximal time interval *t_max_* (a positive constant). Let $t\prime_{s}\left(T\right)$ be *t_max_* if *s* cannot solve task *T*, otherwise it is the time needed to solve *T* by *s*. Let $l\prime_{s}\left(T\right)$ be the positive constant *l_max_* if *s* cannot solve *T*, otherwise it is the number of components of *s* needed to solve task *T* by *s*. The non-negative real-valued reward *r*(*T*) for solving *T* is a positive constant *r_new_* for self-defined previously unsolvable *T*, or user-defined if *T* is an external task solved by *s* (Section 5). The real-valued cost *Cost*(*s*, *TSET*) of solving all tasks in a task set *TSET* through *s* is a real-valued function of: all $l\prime_{s}\left(T\right),t\prime_{s}\left(T\right)$ (for all *T* ∈ *TSET*), *L*(*s*), and Σ*_T ∈TSET_* *r*(*T*). For example, the cost function $Cost(s,\;TSET)=L(s)+\alpha \sum_{T\in TSET}\left[{t^{\prime }}_{s}(T)-r(T)\right]$ encourages compact and fast solvers solving many different tasks with the same components of *s*, where the real-valued positive parameter α weighs space costs against time costs, and *r_new_* should exceed *t_max_* to encourage solutions of novel self-generated tasks, whose cost contributions should be below zero (alternative cost definitions could also take into account energy consumption etc.).

Let us keep an analog of the remaining notation of Section 3.1.2, such as *u_i_*(*t*), *x_i_*(*t*), *r_i_*(*t*), *y_i_*(*t*), *Trace_i_*, *J_i_*(*Trace_i_*). As always, if the environment is unknown and possibly changing over time, to test performance of a new solver *s* on a previous task *T_k_*, only *Trace_k_* is necessary – see Section 3.1.2. As always, let *T*_≤*i*_ denote the set containing all tasks *T*_1_, …, *T_i_* (note that if *T_i_* = *T_k_* for some *k*  < *i* then it will appear only once in *T*_≤*i*_), and let ε > 0 again define what is acceptable progress:

**Algorithm 6.1:** PowerPlay **Framework (Variant II) Explicitly Handling Costs of Solving Tasks**

Initialize *s*_0_ in some way

**for***i:*= 1, 2, …**do**

 Create new global variables *T_i_*  ∈ 𝒯, *s_i_*  ∈ 𝒮, *p_i_* ∈ 𝒫, $c_{i}^{*}$∈ℝ (to be fixed by the end of **repeat**)

 **repeat**

  Let a search algorithm (Section 4.1) set *p_i_*, a new candidate program. Give *p_i_* limited time to do:

  * Task Invention: Unless the user specifies *T_i_* (Section 5), let *p_i_* set *T_i_*.

  * Solver Modification: Let *p_i_* set *s_i_* by computing a modification of *s_i_*_-1_ (Section 3.2).

  * Correctness Demonstration: Let *p_i_* compute *c_i_* := *Cost*(*s_i_*, *T_≤i_*), and $c_{i}^{*}$ := *Cost*(*s_i-1_*, *T_≤i_*)

 **until** $c_{i}^{*}$ -*c_i_* > ϵ (minimal savings of costs such as time/space/etc on all tasks so far)

 Freeze/store forever *p_i_*, *T_i_*, *s_i_*, *c_i_*, $c_{i}^{*}$

**end for**

By Algorithm 6.1, *s_i_* may forget certain abilities of *s_i_*_−1_, provided that the overall performance as measured by *Cost*(*s_i_*, *T*_≤*i*_) has improved, either because a new task became solvable, or previous tasks became solvable more efficiently.

Following Section 3.3, Correctness Demonstration can often be facilitated, for example, by tracking which components of *s_i_* are used for solving which tasks (Section 3.3.2).

To further refine this approach, consider that in phase *i*, the list $L_{i}^{k}$ (defined in Section 3.3.2) contains all previously learned tasks whose solutions depend on *s^k^*. This can be used to determine the current *value* $Val\left(s_{i}^{k}\right)$ of some component *s^k^* of *s:* $\mathrm{Val}\left(s_{i}^{k}\right)=-\sum_{T\in L_{i}^{k}}\mathrm{Cost}\left(s_{i},T_{\leq }i\right)$. It is a simple exercise to invent PowerPlay variants that do not forget valuable components as easily as less valuable ones.

The implementations of Sections 4.1 and 4.3 are easily adapted to the cost-based PowerPlay framework. Compare separate papers (Srivastava et al., 2012b, 2013).

### 6.2 Probabilistic PowerPlay variants

Section 3.1.2 pointed out that in partially observable and/or non-stationary unknown environments Correctness Demonstration must use *Trace_k_* to check whether a new *s_i_* still knows how to solve an earlier task *T_k_*(*k*  < *i*). A less strict variant of PowerPlay, however, will simply make certain assumptions about the probabilistic nature of the environment and the repeatability of trials, assuming that a limited fixed number of interactions with the real world are sufficient to estimate the costs *c*_i_*, *c_i_* in Algorithm 6.1.

Another probabilistic way of softening PowerPlay is to add new tasks without proof that *s* won’t forget solutions to previous tasks, provided Correctness Demonstration can at least show that the probability of forgetting any previous solution is below some real-valued positive constant threshold.

## 7 Discussion

Here I briefly mention illustrative experiments described in detail elsewhere (Srivastava et al., 2012b, 2013) and discuss certain aspects and limitations of PowerPlay. I also discuss related research, in particular, why the present work is of interest despite the recent advent of theoretically optimal universal problem solvers (Section 7.2), and how it can be viewed as a greedy but feasible and sound implementation of the formal theory of creativity (Section 7.4).

### 7.1 Outgrowing trivial tasks – compressing previous solutions

What prevents PowerPlay from inventing trivial tasks forever by extreme modularization, simply allocating a previously unused solver part to each new task, which thus becomes rather quickly verifiable, as its solution does not affect solutions to previous tasks (Section 3.3.3)? On realistic but general architectures such as PCs and RNNs, at least once the upper storage size limit of *s* is reached, PowerPlay will start “compressing” previous solutions, making *s generalize* in the sense that the *same* relatively short piece of code (some part of *s*) helps to solve *different* tasks.

With many computational architectures, this type of compression will start much earlier though, because new tasks solvable by partial reuse of earlier discovered code will often be easier to find than new tasks solvable by previously unused parts of *s*. This also holds for growing architectures with potentially unlimited storage space.

Compare also PowerPlay Variant II of Section 6.1 whose tasks may explicitly require improving the *average* time and space complexity of previous solutions by some minimal value.

In general, however, over time the system will find it more and more difficult to invent novel tasks without forgetting previous solutions, a bit like humans find it harder and harder to learn truly novel behaviors once they are leaving behind the initial rapid exploration phase typical for babies. Experiments with various problem solver architectures (e.g., (Srivastava et al., 2012b, 2013)) help to analyze such effects in detail.

### 7.2 Relation to theoretically optimal universal problem solvers

The new millenium brought universal problem solvers that are theoretically optimal in a certain sense. The fully self-referential (Gödel, 1931) *Gödel machine* (Schmidhuber, 2006b, 2009) may interact with some initially unknown, partially observable environment to maximize future expected utility or reward by solving arbitrary user-defined computational tasks. Its initial algorithm is not hardwired; it can completely rewrite itself without essential limits apart from the limits of computability, but only if a proof searcher embedded within the initial algorithm can first prove that the rewrite is useful, according to the formalized utility function taking into account the limited computational resources. Self-rewrites due to this approach can be shown to be *globally optimal*, relative to Gödel’s well-known fundamental restrictions of provability (Gödel, 1931). To make sure the Gödel machine is at least *asymptotically* optimal even before the first self-rewrite, one may initialize it by Hutter’s non-self-referential but *asymptotically fastest algorithm for all well-defined problems* Hsearch (Hutter, 2002), which uses a hardwired brute force proof searcher and ignores the costs of proof search. Assuming discrete input/output domains *X/Y*  ⊂ B*, a formal problem specification *f: X* → *Y* (say, a functional description of how integers are decomposed into their prime factors), and a particular *x* ∈ *X* (say, an integer to be factorized), Hsearch orders all proofs of an appropriate axiomatic system by size to find programs *q* that for all *z* ∈ *X* provably compute *f* (*z*) within time bound *t_q_*(*z*). Simultaneously it spends most of its time on executing the *q* with the best currently proven time bound *t_q_*(*x*). Hsearch is as fast as the *fastest* algorithm that provably computes *f* (*z*) for all *z* ∈ *X*, save for a constant factor smaller than 1 + ε (arbitrarily small real-valued ε > 0) and an *f* -specific but *x*-independent additive constant (Hutter, 2002). Given some problem, the Gödel machine may decide to replace Hsearch by a faster method suffering less from large constant overhead, but even if it doesn’t, its performance won’t be less than asymptotically optimal.

Why doesn’t everybody use such universal problem solvers for all computational real-world problems? Because most real-world problems are so small that the ominous constant slowdowns (potentially relevant at least before the first self-rewrite) may be large enough to prevent the universal methods from being feasible.

PowerPlay, on the other hand, is designed to incrementally build a *practical* more and more general problem solver that can solve numerous tasks quickly, not in the asymptotic sense, but by exploiting to the max its given particular search algorithm and computational architecture, with all its space and time limitations, including those reflected by constants ignored by the asymptotic optimality notation.

As mentioned in Section 5, however, one must now analyze under which conditions PowerPlay’s self-generated tasks can accelerate the solution to externally generated tasks (compare previous experimental studies of this type (Schmidhuber, 1991a, 1999, 2002; Storck et al., 1995)).

### 7.3 Connection to traditional active learning

Traditional active learning methods (Fedorov, 1972) such as AdaBoost (Freund and Schapire, 1997) have a totally different set-up and purpose: there the user provides a set of samples to be learned, then each new classifier in a series of classifiers focuses on samples badly classified by previous classifiers. Open-ended PowerPlay, however, (1) considers arbitrary computational problems (not necessarily classification tasks); (2) can self-invent all computational tasks; (3) takes into account all computational costs, ordering task candidates by time and space complexity, relative to the present knowledge. There is no need for a pre-defined global set of tasks that each new solver tries to solve better, instead the task set continually grows based on which task is easy to invent and validate, given what is already known.

### 7.4 Greedy implementation of aspects of the formal theory of creativity

The Formal Theory of Creativity (Schmidhuber, 2006a, 2010) considers agents living in initially unknown environments. At any given time, such an agent uses a reinforcement learning (RL) method (Kaelbling et al., 1996) to maximize not only expected future external reward for achieving certain goals, but also *intrinsic* reward for improving an internal model of the environmental responses to its actions, learning to better predict or compress^1^ the growing history of observations influenced by its behavior, thus achieving *wow-effects*, actively learning skills to influence the input stream such that it contains previously unknown but learnable algorithmic regularities. I have argued that the theory explains essential aspects of intelligence including selective attention, curiosity, creativity, science, art, music, humor, e.g., (Schmidhuber, 2006a, 2010). Compare recent related work, e.g., (Salge et al., 2012; Barto, 2013; Dayan, 2013; Nehmzow et al., 2013; Oudeyer et al., 2013).

Like PowerPlay, such a creative agent produces a sequence of self-generated tasks and their solutions, each task still unsolvable before learning, yet becoming solvable after learning. The costs of learning as well as the learning progress are measured, and enter the reward function. Thus, in the absence of external reward for reaching user-defined goals, at any given time the agent is motivated to invent a series of additional tasks that maximize future expected learning progress.

For example, by restricting its input stream to self-generated pairs (*I*, *O*) ∈ ℐ × 𝒪like in Section 3.1.1, and limiting it to predict only *O*, given *I* (instead of also trying to predict future (*I*, *O*) pairs from previous ones, which the general agent would do), there will be a motivation to actively generate a sequence of (*I*, *O*) pairs such that the *O* are first subjectively unpredictable from their *I* but then become predictable with little effort, given the limitations of whatever learning algorithm is used.

Below some of PowerPlay’s apparent drawbacks are listed in light of the above, followed by certain thoughts relativizing those drawbacks.

1. Instead of maximizing future expected reward, PowerPlay is greedy, always trying to find the simplest (easiest to find and validate) task to add to the repertoire, or the simplest way of improving the efficiency or compressibility of previous solutions, instead of looking further ahead, as a universal RL method (Schmidhuber, 2006a, 2010) would do. That is, PowerPlay may potentially sacrifice large long-term gains for small short-term gains: the discovery of many easily solvable tasks may at least temporarily prevent it from learning to solve hard tasks.
  However, on general computational architectures such as RNNs (Section 4.1.2), PowerPlay is expected to soon run out of easy tasks that are not yet solvable, due to the architecture’s limited capacity and its unavoidable generalization effects (many never-tried tasks will become solvable by solutions to the few explicitly tested *T_i_*). Compare Section 7.1.
2. The general creative agent above (Schmidhuber, 2006a, 2010) is motivated to improve performance on the entire history of previous still unsolved tasks, while PowerPlay may discard much of this history, keeping only a selective list of previously solved tasks.
  However, as the system is interacting with its environment, one could store the entire continually growing history, and make sure that 𝒯 always allows for defining the task of better compressing the history so far.
3. PowerPlay as in Section 2 has a binary criterion for adding knowledge (was the new task solvable without forgetting old solutions?), while the general agent (Schmidhuber, 2006a, 2010) uses a more informative information-theoretic measure.
  However, the cost-based PowerPlay framework (Algorithm 6.1) of Section 6 offers similar, more flexible options, rewarding compression or speedup of solutions to previously solved tasks.

On the other hand, drawbacks of previous implementations of formal creativity theory include:

1. Some previous approximative implementations (Schmidhuber, 1991a; Storck et al., 1995) used traditional RL methods (Kaelbling et al., 1996) with theoretically unlimited look-ahead. But those are limited in many ways and not guaranteed to work well in partially observable and/or non-stationary environments where the reward function changes over time. They won’t necessarily generate an optimal sequence of future tasks or experiments.
2. Theoretically optimal implementations (Schmidhuber, 2006a, 2010) are currently still impractical, for reasons similar to those discussed in Section 7.2.

Hence PowerPlay may be viewed as a greedy but feasible implementation of certain basic principles of creativity (Schmidhuber, 2006a, 2010). PowerPlay-based systems are continually motivated to invent new tasks solvable by formerly unknown procedures, or to compress or speed up problem-solving procedures discovered earlier. Unlike previous implementations, PowerPlay extracts from the lifelong experience history a sequence of clearly identified and separated tasks with explicitly recorded solutions. By design it cannot suffer from online learning problems affecting its solver’s performance on previously solved problems.

### 7.5 Beyond algorithmic zero-sum task-invention games

PowerPlay’s most closely related previous task-inventing system is the *dual brain* (Schmidhuber, 1997, 1999, 2002). There, to address the computational costs of learning, and the costs of measuring learning progress, computationally powerful encoders and problem solvers (Schmidhuber, 1997, 2002) are implemented as two very general, co-evolving, symmetric, opposing modules called the *right brain* and the *left brain*. Both are able to influence the construction of self-modifying probabilistic programs written in a universal programing language. An internal storage for temporary computational results of the programs is viewed as part of the changing environment. Each module can suggest experiments or self-invented computational tasks in the form of probabilistic algorithms to be executed, and make predictions about their effects, *betting intrinsic reward* on their outcomes. The opposing module may accept such a bet in a zero-sum game by making a contrary prediction, or reject it. In case of acceptance, the winner is determined by executing the experiment and checking its outcome; the intrinsic reward eventually gets transferred from the surprised loser to the confirmed winner. Both modules try to maximize reward using a rather general RL algorithm (the so-called success-story algorithm SSA (Schmidhuber et al., 1997)) designed for complex stochastic policies (alternative RL algorithms could be plugged in as well). Thus both modules are motivated to discover *novel* tasks exhibiting novel algorithmic patterns/compressibility (=surprising *wow-effects*), where the subjective baseline for novelty is given by what the opponent already knows about the (external or internal) world’s repetitive patterns. Since the execution of any computational or physical action costs something (as it will reduce the cumulative reward per time ratio), both modules are motivated to focus on self-invented tasks that involve those parts of the dynamic world that currently make surprises and learning progress *easy*, to minimize the costs of identifying promising experiments and executing them. The system learns a partly hierarchical structure of more and more complex skills or programs necessary to solve the growing sequence of self-generated tasks, reusing previously acquired simpler skills where this is beneficial. Experimental studies exhibit several sequential developmental stages, with and without external reward (Schmidhuber, 1999, 2002).

However, the *dual brain* system (Schmidhuber, 1999, 2002) did not have a built-in guarantee that it cannot forget previously learned skills, while PowerPlay as in Section 2 does (and the time and space complexity-based variant Algorithm 6.1 of Section 6 can forget only if this improves the average efficiency of previous solutions).

### 7.6 Opposing forces: Improving generalization through compression, breaking generalization through novelty

Two opposing forces are at work in PowerPlay. On the one hand, the system continually tries to improve previously learned skills, by speeding them up, and by compressing the used parameters of the problem solver, reducing its effective size. The compression drive tends to improve generalization performance, according to the principles of *Occam’s Razor* and *Minimum Description Length* (MDL) and *Minimum Message Length* (MML) (Solomonoff, 1964, 1978; Kolmogorov, 1965; Wallace and Boulton, 1968; Rissanen, 1978; Wallace and Freeman, 1987; Li and Vitányi, 1997; Hutter, 2005). On the other hand, the system also continually tries to invent new tasks that break the generalization capabilities of the present solver.

PowerPlay’s time-minimizing search for new tasks automatically manages the trade-off between these opposing forces. Sometimes it is easier (because fewer computational resources are required) to invent and solve a completely new, previously unsolvable problem. Sometimes it is easier to compress (or speed up) solutions to previously invented problems.

### 7.7 Relation to Gödel’s sequence of increasingly powerful axiomatic systems

In 1931, Kurt Gödel showed that for each sufficiently powerful (ω-) consistent axiomatic system there is a statement that must be true but cannot be proven from the axioms through an algorithmic theorem-proving procedure (Gödel, 1931). This unprovable statement can then be added to the axioms, to obtain a more powerful formal theory in which new formerly unprovable theorems become provable, without affecting previously provable theorems.

In a sense, PowerPlay is doing something similar. Assume the architecture of the solver is a universal computer (Gödel, 1931; Church, 1936; Post, 1936; Turing, 1936). Its software *s* can be viewed as a theorem-proving procedure implementing certain enumerable axioms and computable inference rules. PowerPlay continually tries to modify *s* such that the previously proven theorems remain provable within certain time bounds, *and* a new previously unprovable theorem becomes provable.

### 7.8 First illustrative experiments

First experiments with PowerPlay were reported in separate papers (Srivastava et al., 2012b, 2013) (some experiments were also briefly mentioned in the original report (Schmidhuber, 2011)). Standard NNs as well as SLIM RNNs (Schmidhuber, 2012) were used as computational problem-solving architectures. The weights of SLIM RNNs can encode essentially arbitrary computable tasks as well as arbitrary, self-delimiting, halting or non-halting programs solving those tasks (Section 4.1.2). Such programs may affect both environment (through effectors) and internal states encoding abstractions of event sequences. For example, in the experiments a SLIM RNN learned to control a fovea that can be shifted across a visual scene. The sequences of dynamically changing sensory inputs from the fovea contributed to the formation of internal SLIM RNN states, that is, vectors of neural activations encoding possible goals. In open-ended fashion, our PowerPlay-driven NNs learned to become increasingly general solvers of self-invented tasks. Sometimes they added new problem-solving procedures to the growing repertoire. Sometimes they preferred to compress/speed up previously invented skills, depending on what was computationally easiest at this point in time. The NNs also exhibited interesting developmental stages, incrementally moving from apparently simple self-invented problems to more complex ones. Furthermore, it was shown how a PowerPlay-driven SLIM NN automatically self-modularizes (Srivastava et al., 2013), frequently re-using code for previously invented skills, keeping track which connections affect which tasks (Section 3.3.2), always trying to invent novel tasks that can be quickly validated because they do not require too many weight changes affecting too many previous tasks.

## 8 Words of Caution

The behavior of PowerPlay is determined by the nature and the limitations of 𝒯, 𝒮, 𝒫, and its algorithm for searching 𝒫. If 𝒯 includes all computable task descriptions, and both 𝒮 and 𝒫 allow for implementing arbitrary programs, and the search algorithm is a general method for search in program space (Section 4), then there are few limits to what PowerPlay may do (besides the limits of computability (Gödel, 1931)).

It may not be advisable to let a general variant of PowerPlay loose in an uncontrolled situation, e.g., on a multi-computer network on the internet, possibly with access to control of physical devices, and the potential to acquire additional computational and physical resources (Section 3.1.2) through programs executed during PowerPlay. Unlike, say, traditional virus programs, PowerPlay-based systems will continually change in a way hard to predict, incessantly inventing and solving novel, self-generated tasks, only driven by a desire to increase their general problem-solving capacity, perhaps a bit like many humans seek to increase their power once their basic needs are satisfied. This type of artificial curiosity/creativity, however, may conflict with human intentions on occasion. On the other hand, unchecked curiosity may sometimes also be harmful or fatal to the learning system itself (Section 5) – curiosity can kill the cat.

## Conflict of Interest Statement

The authors declare that the research was conducted in the absence of any commercial or financial relationships that could be construed as a potential conflict of interest.

## Footnotes

^1^It is hard to overestimate the cognitive significance of compressing the observation history. For example, consider the video-like image sequence perceived by your brain as you are moving through your office. The natural way of greatly compressing it is to construct an internal 3D model of the office space (here I am generalizing a previous analysis of the emergence of the concept of space (Philipona et al., 2004)). The 3D model allows for re-computing the entire high-resolution video from a compact sequence of very low-dimensional eye coordinates and eye directions. (The model itself typically can be specified by far fewer bits of information than needed to store raw pixel data of a long video.) Even if the 3D model is not quite precise, only relatively few extra bits will be required to encode the observed deviations from the predictions of the model. It seems clear that the enormous compression of sensory inputs achievable through an internal 3D world model is the main reason for the latter’s existence. Data compression also explains the emergence of office space-independent internal representations of *movable* objects such as pens. Many additional examples of data compression in art and science and humor can be found in previous papers (Schmidhuber, 2006a, 2010).
